# Monomeric crystal structure of the vaccine carrier protein CRM_197_ and implications for vaccine development

**DOI:** 10.1107/S2053230X23002364

**Published:** 2023-03-30

**Authors:** D. Travis Gallagher, Natalia Oganesyan, Andrew Lees

**Affiliations:** a National Institute of Standards and Technology, 9600 Gudelsky Drive, Rockville, MD 20850, USA; b Fina Biosolutions LLC, 9430 Key West Avenue, Suite 200, Rockville, MD 20850, USA; Walter Reed Army Institute of Research, USA

**Keywords:** carriers, conjugate vaccines, CRM_197_, diphtheria toxin, domain swapping, toxoids

## Abstract

CRM_197_ is a genetically detoxified mutant of diphtheria toxin (DT) that is widely used as a carrier protein in conjugate vaccines. Protective immune responses to several bacterial diseases are obtained by coupling CRM_197_ to glycans from these pathogens. Wild-type DT has been described in two oligomeric forms: a monomer and a domain-swapped dimer. Their proportions depend on the chemical conditions and especially the pH, with a large kinetic barrier to interconversion. A similar situation occurs in CRM_197_, where the monomer is preferred for vaccine synthesis. Despite 30 years of research and the increasing application of CRM_197_ in conjugate vaccines, until now all of its available crystal structures have been dimeric. Here, CRM_197_ was expressed as a soluble, intracellular protein in an *Escherichia coli* strain engineered to have an oxidative cytoplasm. The purified product, called EcoCRM, remained monomeric throughout crystallization. The structure of monomeric EcoCRM is reported at 2.0 Å resolution with the domain-swapping hinge loop (residues 379–387) in an extended, exposed conformation, similar to monomeric wild-type DT. The structure enables comparisons across expression systems and across oligomeric states, with implications for monomer–dimer interconversion and for the optimization of conjugation.

## Abstract 2

The first crystal structure of the widely used vaccine carrier protein CRM_197_ in its monomeric form reveals features underlying its dimerization and vaccine-conjugation reaction.

## 1. Introduction

Soon after the diphtheria epidemic of 1921, the first modern vaccine was made by treating the diphtheria toxin (DT) from *Corynebacterium diphtheriae* with formaldehyde, creating the diphtheria toxoid vaccine that has been used ever since. Thus inactivated, the protein remains a potent immune stimulator, a feature that has also been exploited to produce several widely used conjugate vaccines against unrelated pathogens through the lysine conjugation of bacterial capsular saccharides. Additional carrier proteins, antigens and linking strategies are under active research and development. CRM_197_ is the G52E mutant of DT, which renders it catalytically inactive and thus nontoxic, without the lysine adducts and heterogeneity that result from formaldehyde treatment (Giannini *et al.*, 1984 ▸; Bröker *et al.*, 2011 ▸; Malito *et al.*, 2012 ▸). This innovation gained FDA approval in 2000 and is currently the basis of many conjugate vaccines, although CRM_197_ has not replaced the toxoid in the widely used diphtheria–tetanus–pertussis vaccines. CRM_197_ serves as a conjugate carrier protein in vaccines against *Haemophilus influenzae* type b, *Streptococcus pneumoniae*, *Salmonella* Typhi and meningococcal diseases made by coupling CRM_197_ to components from those pathogens. Tetanus toxoid, diphtheria toxoid and CRM_197_ are the most widely used vaccine carrier proteins. CRM_197_ is the carrier protein in the *S. pneumoniae* vaccine Prevnar, which is one of the most widely distributed vaccines.

Extensive studies of the physical and chemical properties of CRM_197_ have provided data on its biophysical (Porro *et al.*, 1980 ▸; Hickey *et al.*, 2018 ▸; Bravo-Bautista *et al.*, 2019 ▸) and conjugate-synthesis behavior (Crotti *et al.*, 2014 ▸; Möginger *et al.*, 2016 ▸; Jaffe *et al.*, 2019 ▸). CRM_197_ was originally produced in *C. diphtheriae*, the biological source of diphtheria toxin. More recently, it has been expressed in *Pseudomonas fluorescens* and in the periplasm of *Escherichia coli*. CRM_197_ has two disulfide bonds, so cytoplasmic expression in unmodified *E. coli* tends to result in inclusion bodies. A recently developed *E. coli* strain with glutathione reductase deleted (Δ*gor*) produces soluble, intracellular, properly folded, vaccine-competent, recombinant CRM_197_, designated EcoCRM (Oganesyan & Lees, 2018 ▸).

To further characterize EcoCRM and to assess its similarity to CRM_197_ produced using other expression systems, we determined its crystal structure (PDB entry 7rrw) and found that it was monomeric. This was not expected, since all four previous crystal structures of CRM_197_, along with most structures of wild-type DT, reveal a domain-swapped dimer in which the C-terminal domain (residues 390–535) is exchanged with an adjacent molecule (Carroll *et al.*, 1986 ▸; Bennett *et al.*, 1994 ▸; Bennett & Eisenberg, 1994 ▸). For vaccine applications, among the theoretically conjugable primary amines (39 lysines plus the N-terminus), a subset have been reported to be preferentially loaded by conjugation (Möginger *et al.*, 2016 ▸; Kuttel *et al.*, 2021 ▸). Because the oligomeric state of CRM_197_ may affect its conjugation and vaccine properties, structural analysis of the differences between the monomeric and dimeric forms may be useful in creating more homogeneous conjugates and more effective vaccines.

## 2. Materials and methods

### 2.1. Macromolecule production

The CRM_197_ gene was optimized for expression in *E. coli* and synthesized by DNA2.0 (now ATUM, Newark, California, USA). The gene was inserted into pTac24, a vector based on pET-24a (Novagen) in which the T7 promoter is replaced by a synthetic fragment containing the *tac* promoter. The resulting plasmid was transformed into BL21 Δ*gor* cells (Oganesyan & Lees, 2018 ▸) by electroporation for expression (Table 1 ▸). Transformed cell colonies were selected based on kanamycin resistance, and expression was induced by isopropyl β-d-1-thiogalactopyranoside at 0.5 m*M*. SDS–PAGE was used to confirm CRM_197_ expression at a molecular weight of 58 kDa, and Western blots were performed to confirm the identity of CRM_197_. The cells were stored as glycerol stocks at −70°C.

For protein production, a seed culture was prepared by inoculating 1 mL glycerol stock into 50 mL MDG medium (Studier, 2014 ▸) in a 250 mL baffled flask and grown overnight in a 37°C shaker incubator at 250 rev min^−1^. The seed culture was used to inoculate fed-batch fermentation in 3 L medium containing kanamycin in a 5 L New Brunswick fermenter. Fermentation was controlled by a Lab Owl Bio­reactor Control System. The cells were harvested by centrifugation. Approximately 250 g of cell paste was obtained per litre of fermentation culture. The paste was resuspended in cold lysis buffer and the cells were opened by homogenization. The resulting cell lysate was clarified by centrifugation at 4°C and by filtration with a 0.45 µm PES filter. Filtrate containing approximately 3 g L^−1^ soluble CRM_197_ was loaded onto an anion-exchange column and the eluent was then applied onto a cation-exchange column for polishing. The resulting eluent containing the purified protein was then concentrated and diafiltrated into 20 m*M* HEPES pH 8.0 by tangential flow filtration. Sucrose and Tween 80 were added to 10% and 0.0055%, respectively. Testing using nonreduced and reduced SDS–PAGE, size-exclusion chromatography, Endosafe (Charles River Laboratories) and host protein analysis (Cygnus Technologies) showed that the protein was over 98% monomer (a certificate of analysis is available on request).

### 2.2. Crystallization

20 mg purified protein was dialyzed into 20 m*M* HEPES pH 8.0, concentrated to 20 mg mL^−1^ and screened for crystallization by mixing 2 µL with a similar volume of various solutions of salts and polymers and then incubating the mixture in equilibrium with the solution in a sealed chamber. Static light scattering indicated that the sample was monomeric (Supplementary Fig. S1). About 300 conditions were screened; two of these yielded crystals. Fine adjustments to the conditions changed the initial spherules into branching clusters of thin plates and led to the optimized conditions given in Table 2 ▸.

### 2.3. Data collection and processing

A crystal of 20 × 100 × 150 µm in size was dunked into cryoprotectant for 2 s and then cryocooled by plunging it into liquid nitrogen for data collection on beamline 23-ID-B at the Advanced Protein Source (APS), Argonne National Laboratory. Data were integrated and scaled using programs from the *CCP*4 crystallographic suite (Winn *et al.*, 2011 ▸). See Table 3 ▸ for diffraction statistics.

### 2.4. Structure solution, refinement and analysis

The monomeric CRM_197_ structure was solved by molecular replacement with *Phaser* (Storoni *et al.*, 2004 ▸) using a monomeric model based on PDB entry 5i82 (Mishra *et al.*, 2018 ▸). The structure was subjected to ten rounds of refinement, with each round comprising map inspection, model adjustments, iterative global minimization using *REFMAC* (Murshudov *et al.*, 2011 ▸) and calculation of a new map. *PyMOL* (https://pymol.org) was used for all map inspection and model building and to prepare figures. Statistics for the refinement and final model are given in Table 4 ▸. Solvent-accessible surface area (SASA) calculations used the *CCP*4 program *AREAIMOL* with a probe radius of 2.5 Å. For the accessibilities of lysines, the SASA values of the five side-chain atoms were summed.

## 3. Results and discussion

Three internal zones are missing due to disorder. The first zone is residues 30–33, a surface loop near the active site, and the second is residues 39–49, which form the active-site loop that is only fully ordered in wild-type structures that include a substrate analog. The G52E mutation site is well ordered in the present structure. The third disordered zone comprises residues 187–200. This region at the junction of the catalytic and membrane-fusion domains of the protein has never been observed crystallographically; however, the disulfide 186–201 bridges the missing zone and connects the domains. This disulfide in CRM_197_ was the subject of a recent study (Carboni *et al.*, 2022 ▸) and structure (PDB entry 7o4w). Both this disulfide and the second disulfide (461–471) are well ordered in the present structure.

Most reported DT structures are dimeric, with only one unique DT structure that is monomeric, PDB entry 1mdt; PDB entry 1f0l is the same structure at higher resolution. PDB entry 7rrw superposes onto PDB entry 1f0l with a root-mean-square deviation (r.m.s.d.) of 1.2 Å for all C^α^ atoms. Five CRM_197_ structures have now been reported: the present monomeric structure, two dimeric structures resulting from expression in *E. coli* (PDB entries 5i82 and 7o4w) and two dimeric structures resulting from expression in *P. fluorescens* (PDB entries 4ae0 and 4ae1). Mishra *et al.* (2018 ▸) reported on the similarity between PDB entries 5i82 and 4ae0; the *P. fluorescens*-produced protein used to obtain these structures is also used in Vaxneuvance, an FDA-approved pneumo­coccal conjugate vaccine. Due to this similarity, the present monomeric CRM_197_ structure is compared primarily with PDB entry 5i82. The C^α^ r.m.s.d. between PDB entry 7rrw and a monomer-like construct formed from chain *A* residues 1–378 and chain *B* residues 388–535 of PDB entry 5i82 (*i.e.* omitting the hinge loop) is 0.91 Å. Adding the hinge loop to the calculation increases the r.m.s.d. to 1.37 A due to its different structure in the monomer versus the dimer. Preserving pharmaceutical homogeneity requires methods to control the oligomeric state of CRM_197_ from expression to vaccine administration. It is not clear whether the observed variability results from differences in expression, purification or crystallization. Based on comparing the pH across known structures (including during protein preparation), it is likely that maintaining a high pH and avoiding phosphate were important in maintaining the monomeric state observed in the present report.

**Figure 1:**
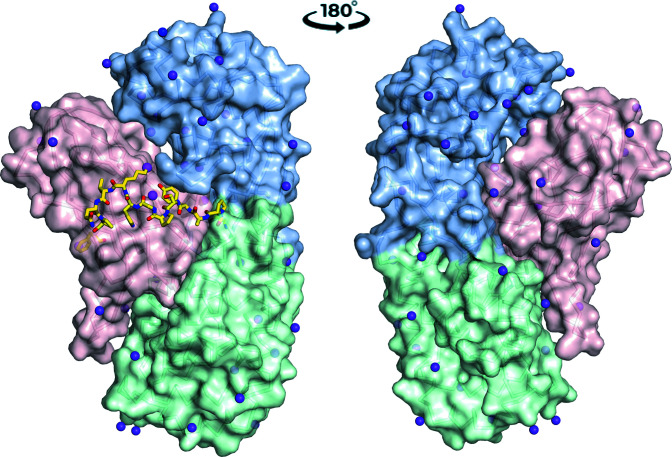
Overall structure of monomeric CRM_197_ in two views, showing its three domains with colored surfaces. The 40 primary amines (lysines plus the N-terminus) are shown as spheres. The N-terminal sphere and N-terminal catalytic domain are colored blue, the second domain is colored green and the C-terminal receptor-binding domain is colored pink. The hinge loop that connects domains 2 and 3 and that rearranges to form the dimer is shown in stick representation. The ɛ-amines of Lys385 (within the hinge) and Lys419 (behind the hinge) are colored magenta.

**Figure 2:**
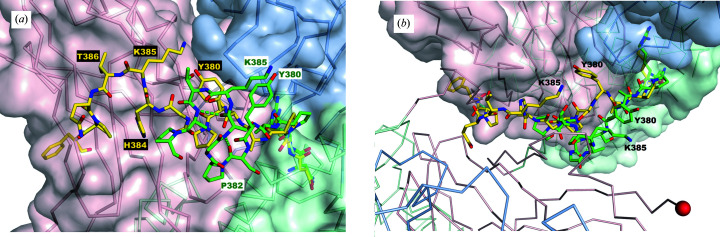
Close-up of the dimerization hinge loop in CRM_197_, showing its conformational change from the monomer (yellow; PDB entry 7rrw) to the dimer (green; PDB entry 5i82). Some side chains are unobserved in PDB entry 5i82. (*a*) Green dashed lines show the two intra-chain hydrogen bonds that form in the dimeric conformation: Gly383–Pro388 and His384–Gln387. (*b*) View from above (the direction of view is approximately along the dimer axis). The second protomer is added outside the surface of the first protomer. The red sphere indicates a C-terminus.

In a domain-swapped dimer, the connecting loop or hinge is the only part that changes its conformation. Both it and its contacting residues change their local environment. In theory there are three distinct structural states: a closed monomer, a transient open monomer and a dimer (Liu & Eisenberg, 2002 ▸). In the present case, the hinge consists of residues 379–387 (see Figs. 1 ▸ and 2 ▸). The hinge connects the large module comprising the N-terminal (catalytic and transmembrane) domains to the C-terminal (receptor-binding) domain. Fig. 2 ▸ superposes the hinge loops in the monomer and dimer, showing the different conformations. The dimer conformation gains two positive-φ residues, Gly383 and Lys385, and is more compact; the C^α^ distance between Tyr380 and Pro388 decreases from 16.0 to 9.8 Å. The shorter loop is consistent with the dimerization mechanism suggested by Shahid *et al.* (2021 ▸). Another feature that would be predicted to stabilize the dimeric conformation of CRM_197_ is that the hinge goes from having zero to two main-chain hydrogen bonds (Fig. 2 ▸).

Mapping conjugation efficiency across the amine sites has found that a subset of lysines dominate, including Lys95, Lys103, Lys212, Lys221, Lys242, Lys236, Lys498 and Lys526 (Möginger *et al.*, 2016 ▸; Kuttel *et al.*, 2021 ▸). Most of these are highly exposed surface sites. In CRM_197_, the side chain of Lys385 in the hinge loop is more solvent-exposed in the monomeric structure (SASA of 91.6 Å^2^) than in the dimer (SASA of 2.4 Å^2^). Lys419 also undergoes a large change in its environment, although its solvent exposure is near zero in both crystal structures.

We have described the first monomeric crystal structure of the vaccine carrier protein CRM_197_ and compared it with its dimeric precedents. Comparisons show that the CRM_197_ structure is largely conserved across changes in expression host, inactivating mutations and oligomeric states. Observed differences in the hinge loop will inform efforts to understand the energetics of dimerization and thus to control the oligomeric state, while differences in the environments of Lys385 and Lys419 may affect conjugation efficiencies at these sites.

## Supplementary Material

PDB reference: monomeric CRM_197_, 7rrw

Supplementary Figure. DOI: 10.1107/S2053230X23002364/jg5007sup1.pdf

## Figures and Tables

**Table 1:** Macromolecule-production information

Source organism	*Corynebacterium diphtheriae*
DNA source	Synthesized by ATUM (DNA2.0)
Expression vector	pTac24
Expression host	*Escherichia coli* BL21
Complete amino-acid sequence of the construct	MGADDVVDSSKSFVMENFSSYHGTKPGYVDSIQKGIQKPKSGTQGNYDDDWKEFYSTDNKYDAAGYSVDNENPLSGKAGGVVKVTYPGLTKVLALKVDNAETIKKELGLSLTEPLMEQVGTEEFIKRFGDGASRVVLSLPFAEGSSSVEYINNWEQAKALSVELEINFETRGKRGQDAMYEYMAQACAGNRVRRSVGSSLSCINLDWDVIRDKTKTKIESLKEHGPIKNKMSESPNKTVSEEKAKQYLEEFHQTALEHPELSELKTVTGTNPVFAGANYAAWAVNVAQVIDSETADNLEKTTAALSILPGIGSVMGIADGAVHHNTEEIVAQSIALSSLMVAQAIPLVGELVDIGFAAYNFVESIINLFQVVHNSYNRPAYSPGHKTQPFLHDGYAVSWNTVEDSIIRTGFQGESGHDIKITAENTPLPIAGVLLPTIPGKLDVNKSKTHISVNGRKIRMRCRAIDGDVTFCRPKSPVYVGNGVHANLHVAFHRSSSEKIHSNEISSDSIGVLGYQKTVDHTKVNSKLSLFFEIKS

**Table 2:** Crystallization

Method	Hanging-drop vapor diffusion
Plate type	VDX, Hampton Research
Temperature (K)	295
Protein concentration (mg mL^−1^)	20
Buffer composition of protein solution	20 m*M* sodium HEPES pH 8.0
Composition of reservoir solution	12%(*w*/*v*) PEG 10 000, 40 m*M* calcium acetate pH 8.0
Volume and ratio of drop	3.8 µL protein solution plus 5 µL reservoir solution (3:4)
Volume of reservoir (µL)	400
Cryoprotectant	18% glycerol + 82% reservoir solution

**Table 3:** Data collection and processing Values in parentheses are for the outer shell.

Diffraction source	Beamline 23-ID-B, APS
Wavelength (Å)	1.0332
Temperature (K)	100
Detector	Dectris EIGER X 16M
Crystal-to-detector distance (mm)	250.0
Rotation range per image (°)	0.5
Total rotation range (°)	250
Exposure time per image (s)	0.5
Space group	*P*2_1_
*a*, *b*, *c* (Å)	59.02, 81.67, 71.76
α, β, γ (°)	90, 110.87, 90
Mosaicity (°)	0.22
Resolution range (Å)	45.71–1.65 (1.69–1.65)
Total No. of reflections	326822 (11771)
No. of unique reflections	74330 (4284)
Completeness (%)	97.3 (77.0)
Multiplicity	4.4 (2.7)
〈*I*/σ(*I*)〉	8.7 (0.5)†
*R*_p.i.m._	0.055 (1.420)†
Overall *B* factor from Wilson plot (Å^2^)	29.5

† The low signal-to-noise ratio for the outer shell of data indicates that it is weak and of little informational value, consistent with the finding that refinement against data beyond 2.0 Å resolution failed to decrease *R*_free_. For this reason, refinement used data to 2.0 Å resolution only. The value of 〈*I*/σ(*I*)〉 decreases to 2.0 at about 1.9 Å resolution.

**Table 4:** Structure solution and refinement of monomeric CRM_197_ (PDB entry 7rrw) Values in parentheses are for the outer shell.

Resolution range (Å)	16.00–2.00 (2.051–2.000)
Completeness (%)	99.1
σ Cutoff	*F* > 0.000σ(*F*)
No. of reflections, working set	40508 (2994)
No. of reflections, test set	2222 (154)
Final *R*_cryst_	0.178 (0.195)
Final *R*_free_	0.217 (0.245)
Cruickshank DPI	0.087
No. of non-H atoms	
Total	4316
Protein	3884
Solvent	424
R.m.s. deviations	
Bond lengths (Å)	0.009
Angles (°)	1.545
Average *B* factors (Å^2^)	
Protein	27.5
Water	45.1
Ramachandran plot	
Most favored (%)	93.2
Allowed (%)	6.8
