# Supplementary material for: Monomeric crystal structure of the vaccine carrier protein CRM197 and implications for vaccine development
Source: Acta Crystallogr F Struct Biol Commun. 2023 Mar 30;79(Pt 4):82–6. doi: 10.1107/S2053230X23002364 (PMC10071833; doi:10.1107/S2053230X23002364)
Supplement: Supplementary file 1 [file f-79-00082-sup1.pdf]

STRUCTURAL BIOLOGY COMMUNICATIONS

Volume 79 (2023)

Supporting information for article:

Monomeric crystal structure of the vaccine carrier protein CRM_{197} and implications for vaccine development

D. Travis Gallagher, Natalia Oganesyan and Andrew Lees

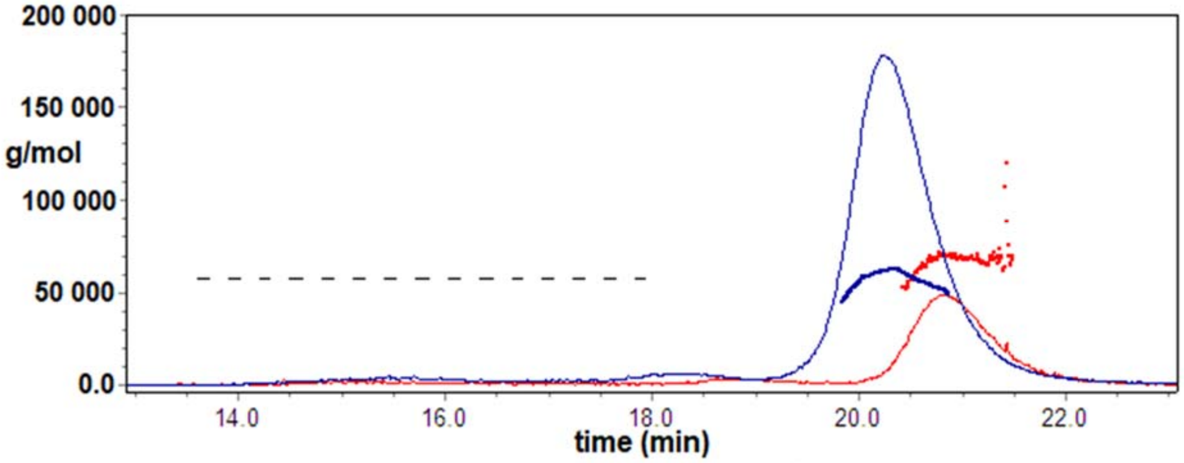

Figure S1 Superposed chromatograms showing the scattered light intensity (Rayleigh ratio) over size-exclusion chromatography (SEC) elution time for two runs of the crystallized CRM197 sample on different days. Also shown are the calculated molar mass values (thick lines, units of g/mol) across the two peaks, which both correspond closely to the predicted value of ~58 kDa (dashed line) for monomeric CRM197. SEC-coupled static multi-angle static light scattering was used to measure the native molar mass in solution. This method uses refractive index as a proxy for concentration, which along with Rayleigh ratio can be used to calculate the MW for pure species. The SEC column used was a Tosoh TSKgel SuperSW2000, the running buffer was 50 mM NaCl, 20 mM His, pH 7.5, and the instrument was a Mini Dawn running ASTRA software from Wyatt Technologies
